# Surveillance on Healthcare Workers During the First Wave of SARS-CoV-2 Pandemic in Italy: The Experience of a Tertiary Care Pediatric Hospital

**DOI:** 10.3389/fpubh.2021.644702

**Published:** 2021-07-26

**Authors:** Valentina Guarnieri, Maria Moriondo, Mattia Giovannini, Lorenzo Lodi, Silvia Ricci, Laura Pisano, Paola Barbacci, Costanza Bini, Giuseppe Indolfi, Alberto Zanobini, Chiara Azzari

**Affiliations:** ^1^Meyer Children's University Hospital, Florence, Italy; ^2^Department of Health Sciences, University of Florence, Florence, Italy; ^3^Department of Neurofarba, University of Florence, Florence, Italy

**Keywords:** surveillance, SARS-C0V-2, COVID-19, healthcare worker, nasopharangeal swabs, serologic tests

## Abstract

Healthcare workers (HCWs) play a central role in handling the ongoing coronavirus disease 2019 (COVID-19) pandemic. Monitoring HCWs, both symptomatic and asymptomatic, through screening programs, are critical to avoid the spread of severe acute respiratory syndrome coronavirus 2 (SARS-CoV-2) infection in the hospital environment to rapidly identify and isolate infected individuals and to allow their prompt return to work as soon as necessary. We aim to describe our healthcare surveillance experience (April 2–May 6, 2020) based on a combined screening consisting of real-time PCR (RT-PCR) on nasopharyngeal (NP) swabs and rapid serologic tests (RST) for SARS-CoV-2 in all HCWs of Meyer Children's University Hospital in Florence. Among the analyzed workers, 13/1690 (0.8%), all of them without clinical manifestations, was found positive for SARS-CoV-2 by using RT-PCR on NP swab: 8/1472 (0.5%) were found positive during the screening, 1/188 (0.5%) during contact with a positive individual (*p* > 0.05 vs. screening group), while 4/30 (13.3%) were found positive on the day of re-admission at work after an influenza-like-illness (*p* < 0.05). Concerning working areas, the majority of RT-PCR positivity (12/13) and serologic positivity (34/42) was found in non-COVID-19 dedicated areas (*p* > 0.05 vs. COVID-19 dedicated areas). No cases were registered among non-patients-facing workers (*p* = 0.04 vs. patient-facing group). Nurses and residents represented, respectively, the working role with the highest and lowest percentage of RT-PCR positivity. In conclusion, accurate surveillance is essential to reduce virus spread among HCWs, patients, and the community and to limit the shortage of skilled professionals. The implementation of the surveillance system through an efficient screening program was offered to all professionals, regardless of the presence of clinical manifestations and the level of working exposure risk, maybe wise and relevant.

## Introduction

As of June 12, 2021, severe acute respiratory syndrome coronavirus 2 (SARS-CoV-2) infection, which is responsible for coronavirus disease 2019 (COVID-19), has caused almost four million deaths, becoming a major global life threat. Italy has experienced 214,457 cases of COVID-19, and 29,684 deaths up to May 6, 2020 (1–3).

Most patients are with or without mild clinical manifestations (4–8), and the transmission of SARS-CoV-2 from asymptomatic individuals raises the occurrence of COVID-19 and the consequent need for skilled healthcare staff working in the care of COVID-19 patients.

The infection among healthcare workers (HCWs) results in a critical factor for two main reasons: first, a positive worker may represent serious harm becoming a source of infection for patients and colleagues; second, HCWs play a central role in handling the ongoing pandemic; therefore, their absence from work leads to a shortage of skilled HCWs (9). Hence, the risk of losing them threatens the management of the hospitals during the ongoing COVID-19 pandemic worldwide. Furthermore, HCWs are crucial for patient care during the COVID-19 pandemic to deal with routine hospital care needs as well.

Sources of HCWs infections may be multiple: direct contact with infected patients or other HCWs in COVID-19 wards or hospitals and community transmission, e.g., *via* infected family members and other close contacts.

The community transmission is addressed by public health measures, such as social distancing, appropriate use of masks, and frequent hand cleaning (10). In addition, to reduce nosocomial transmission, measures of prevention, including disinfection of the environment and hands, appropriate use of personal protective equipment, social distancing, and control with surveillance programs are essential (11). Specifically, monitoring HCWs, both symptomatic and asymptomatic, during the COVID-19 pandemic through screening programs, are critical to guarantee the absence of SARS-CoV-2 infection among HCWs to identify and isolate infected ones rapidly and to allow their prompt return to work as soon as possible (12).

The management of SARS-CoV-2 risk among healthcare staff can rely on many national and international recommendations (including those from the Centers for Disease Control and Prevention, CDC and WHO) (13–16); however, these suggestions need to be converted into practical approaches considering characteristics of the hospital facility and available financial resources.

In this study, we share our hospital surveillance program experience carried out according to the local policy during the first wave of the COVID-19 pandemic in Italy.

We aimed to monitor HCWs with all methodologies available at that time and to analyze different risks of exposure in hospital wards and among different working role categories. This observational cross-sectional study has provided valuable evidence, which was used by our Health Directorate, to set up and adjust subsequent HCWs surveillance programs over time.

## Methods

### Study Population

A combined screening, including real-time PCR (RT-PCR) on nasopharyngeal (NP) swabs and a rapid serologic test (RST) for SARS-CoV-2, was performed in the period April 2–May 6, 2020, on 1,472 workers (including administrative staff) at Meyer Children's University Hospital in Florence, where a significant number of Italian pediatric patients with COVID-19 were admitted at that time.

Furthermore, RT-PCR on NP swabs was routinely performed on all workers before re-admissions at work after recovering from an acute influenza-like illness (*n* = 30) together with an RST test performed to include them into the screening program. Finally, in positive cases among workers or patients, in addition to the combined screening mentioned above, strict monitoring of contacts was set up immediately through RT-PCR on NP swabs (*n* = 188). In total, 1,690 RT-PCR tests on NP swabs and 1,502 RST were analyzed on 1,502 HCWs.

Prevention protocols adopted by the hospital during the period of study were in accordance with the existing guidelines by WHO and CDC and similar to policies adopted by other Italian and international hospitals in the same period (17–20). Thus, in addition to structural and organizational changes (establishment of COVID-19 dedicated areas and care pathways), workers were educated to follow behavioral rules, including hygienic protocols, correct use of personal protective equipment, social distancing among colleagues, and symptoms and contacts self-monitoring.

Furthermore, mass temperature body screening of HCWs was performed routinely at the entrance of the hospital with a thermal scanner, and frequent hand cleaning was considered mandatory inside the hospital (21).

Professionals who tested positive with SARS-CoV-2 though RT-PCR swabs were immediately excluded from work, remained at home for isolation, and an investigation of their contacts was promptly implemented. Workers with acute influenza-like illness were isolated as well, and a negative RT-PCR swab was considered compulsory to be readmitted to work.

The risk of exposure was categorized into three classes based on the working area: (1) professionals working in COVID-19 areas (i.e., COVID-19 wards, emergency unit, infectious disease unit, and laboratory), (2) professionals working in non-COVID-19 dedicated clinical areas, and (3) professionals working in non-clinical areas. Specific working areas have been analyzed separately to figure out the occurrence of diagnostic tests positivity in each category.

Furthermore, we considered three different working role categories: (1) patient-facing workers; (2) non-patients facing personnel with high SARS-CoV-2 exposure risk (i.e., biologists, chemists, and technicians), and (3) non-patients facing professionals with low risk of exposure to SARS-CoV-2 (i.e., administrative staff, physicists, engineers, and pharmacists). Specific working roles were analyzed separately to figure out the occurrence of diagnostic tests positivity in every category.

### Laboratory Analyses

RT-PCR was performed with a homemade method based on primers and probes described by the CDC (22). Positive cases were confirmed by a second NP swab.

Specifically, RNA was extracted from 200 μl of respiratory specimens by using the MagCore Viral Nucleic Acid2 Extraction Kit 96 Preps according to the instructions of the manufacturer (RBC, Bioscience, Korea). About 60 μl elution volume was obtained. RT-PCR amplification of N genes of SARS-CoV-2 was performed by using the TaqPath 1-Step RT-qPCR Master Mix, CG (Thermo Fisher Scientific, Massachusetts, United States). Each 20 μl reaction contained 5 μl of 4X Master Mix, 0.5 μl of 5 μmol/L probes, 0.5 μl each of 20 μmol/L forward and reverse primers, 8.5 μl of nuclease-free water, and 5 μl of nucleic acid extract. We conducted amplification in 96-well plates on an Applied Biosystems 7,500 Fast Real-Time PCR Instrument (Thermo Fisher Scientific, Massachusetts, United States). Thermocycling conditions consisted of 2 min at 25°C for uracil-DNA glycosylase incubation, 15 min at 50°C for reverse transcription, 2 min at 95°C for activation of the Taq enzyme, and 45 cycles of 3 s at 95°C, and 30 s at 55°C. A positive test result was defined as an exponential fluorescent curve that crossed the threshold within 40 cycles [threshold cycle (Ct) <40].

The presence of SARS-COV-2 antibodies (IgG and IgM) was evaluated through an RST (Zhejiang Orient Gene Covid-19 IgG/IgM Rapid Test Cassette, Biotech Co., LTD, Zhejiang, China) according to the instructions of the manufacturer (23).

### Data and Statistical Analysis

Data and statistical analysis were processed using the SPSS statistical package. Descriptive statistics (frequency of distributions) were performed on subsamples of the study population; a Chi-squared test was used to evaluate the association between SARS-CoV-2 infection different exposures, i.e., among different working areas and roles. A statistically significant difference was defined by a *p* < 0.05.

## Results

Characteristics of workers cohort during the surveillance program according to RT-PCR on NP swabs and RST positivity are shown in Table 1.

**Table 1:** Characteristics of the cohort of workers, during the surveillance program (April 2–May 6, 2020) according to real-time PCR (RT-PCR) on nasopharyngeal (NP) swabs and rapid serological test (RST) results.

	***N* (%) (*N* = 1,690)**	**RT-PCR positivity**	***N* (%) (*N* = 1,502)**	**Serological test positivity (RST)**	***P*** **values**	
					RT-PCR	RST
**Median age (years)**	42					
*Interquartile range*	33–51					
**Sex**						
*Female*	1,263 (74.7%)	12/1,262 (0.9%)	1,112 (74.0%)	37/1,112 (3.3%)	–	–
*Male*	427 (25.3%)	1/427 (0.2%)	390 (26.0%)	5/390 (1.3%)	>0.05	<0.05
**Total**	1,690	13/1,690 (0.8%)	1,502	42/150 (2.8%)		
**Aim of the surveillance program**						
*Screening*	1,472 (87.1%)	8/1,472 (0.5%)	1,472 (98.0%)	37/1,472 (2.5%)	–	–
*Contacts*^α^	188 (11.1%)	1/188 (0.5%)			>0.05	
*Re-admission at work after acute influenza-like illness*	30 (1.7%)	4/30 (13.3%)	30 (2.0%)	5/30 (16.6%)	<0.05	<0.05
**Working area**						
*COVID-19 dedicated clinical areas*^β^	351 (20.8%)	0/351 (0.0%)	325 (21.6%)	5/325 (1.5%)	–	–
*Non-COVID-19 dedicated clinical areas*	1,147 (67.9%)	12/1,147 (1.0%)	990 (65.9%)	34/990 (3.4%)	>0.05	>0.05
*Non-clinical areas*	192 (11.4%)	1/192 (0.5%)	187 (12.5%)	3/187 (1.6%)	>0.05	>0.05
**Working Role**						
*Patients facing professionals*	1,416 (83.8%)	13/1,416 (0.9%)	1,233 (82.1%)	39/1,233 (3.2%)		
*Non-patients facing professionals (laboratory)*^γ^	158 (9.3%)	0/158 (0.0%)	155 (10.3%)	3/155 (1.9%)	>0.05	>0.05
*Non-patients facing professionals (other members of the staff)*^δ^	116 (6.9%)	0/116 (0.0%)	114 (7.6%)	0/114 (0.0%)	>0.05	>0.05

α *Professionals tested because of contact with positive cases*.

β *COVID-19 dedicated areas: COVID-19-ward, Emergency department, Infectious disease Unit or Laboratory*.

γ *Non-patients facing health professionals with high COVID-19 exposure risk: biologists, chemicals and technicians*.

δ *Non-patients facing professionals with low risk of COVID-19 exposure: administrative staff, physicists, engineers and pharmacists*.

Among all workers analyzed, 13/1,690 (0.8%, all of them without clinical manifestations) were found positive by using the RT-PCR test on NP swab: 8/1,472 (0.5%) were found during the screening, 1/188 (0.5%) during the contact with a positive case (*p* > 0.05 vs. screening group), while 4/30 (13.3%) were found positive on the day of re-admission at work after an acute influenza-like-illness (*p* < 0.05 vs. screening group).

As for RST, 42/1,502 (2.8%) workers were found positive for IgM, IgG, or both against SARS-CoV-2: 37/1,472 (2.5%) were detected during the screening program, while 5/30 (16.6%) workers were found on the day of the re-admission after an acute illness. Among the 13 workers who were positive for SARS-CoV-2 by using the RT-PCR test on NP swab, only 7/13 (53.8%) were found positive by using the RST (five during the combined screening and two at the re-admission at work).

Concerning working areas, among workers in COVID-19 areas, no RT-PCR positivity was registered with the lowest serological positivity rate (5/325, 1.5%), while the majority of RT-PCR positivity (12/13, 92.3%) and IgM-IgG positivity (34/42, 80.9%) were found in non-COVID-19 dedicated areas (*p* > 0.05 vs. COVID-19 dedicated areas).

Regarding working roles, patient-facing professionals resulted being the 83.8% of this study sample, while non-patient-facing professionals were divided by the risk of SARS CoV-2 exposure in biologists, chemists, and technicians with high exposure risk (9.3%), whereas administrative staff, physicists, engineers, and pharmacists with low exposure risk (6.9%).

The percentage of positivity (either at the NP swab or the RST) was 2.8% (48/1690) with 45 cases that occurred in the patient-facing workers and no case among non-patients facing workers with low exposure risk (*p* < 0.05 vs. patient-facing group).

**Figure 1:**
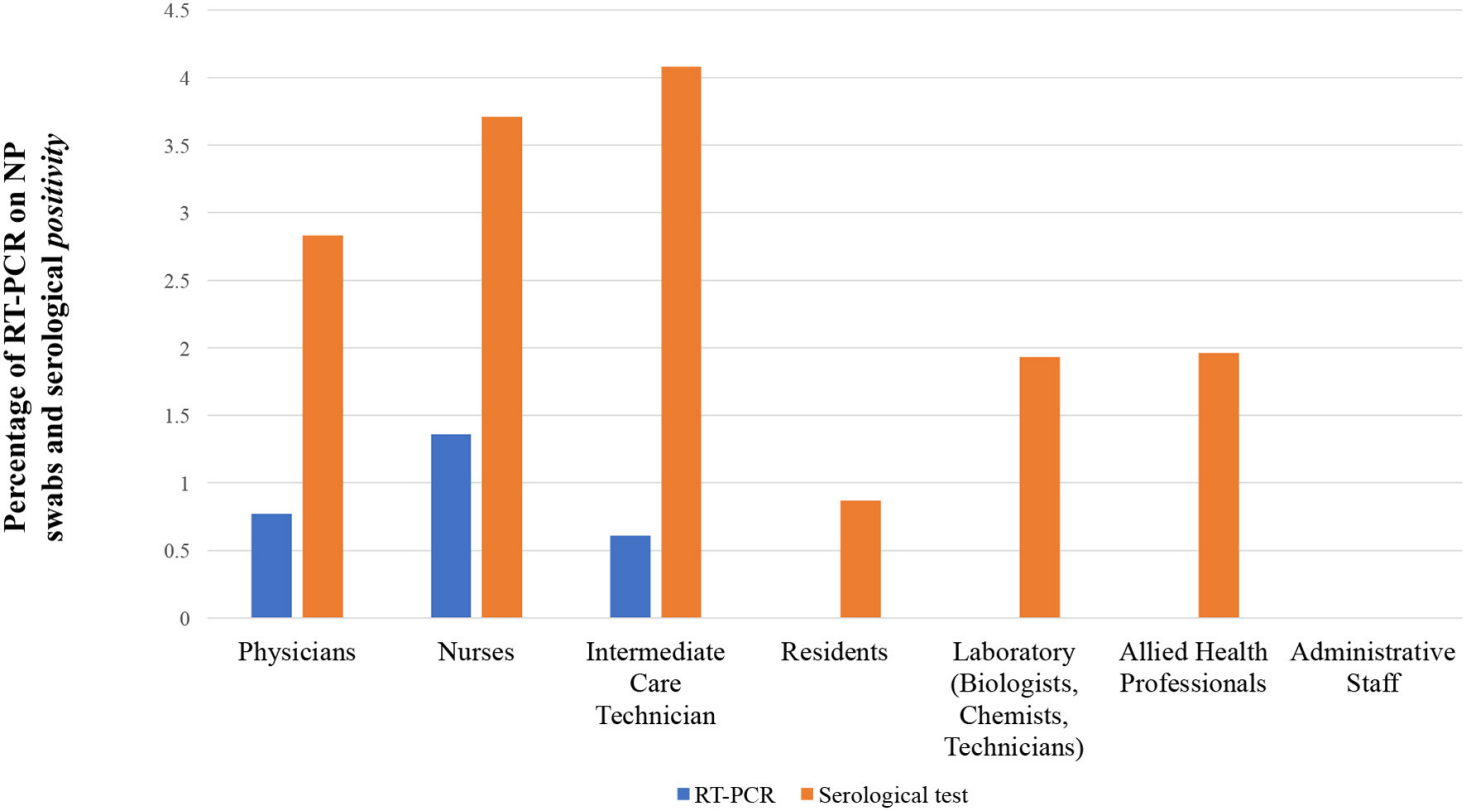
Percentage of HCWs found positive with RT-PCR on NP swabs or rapid antibody test on serological samples (IgG, IgM or both) divided by specific working roles.

Concerning single working roles, nurses represent the working role with the highest percentage of RT-PCR positivity on NP swabs (9/663 nurses, 1.4%) accounting for more than 69% of total positive cases identified. On the contrary, residents resulted in being the less affected working role within the category of patient-facing health providers with no RT-PCR positivity on NP swab (Figure 1).

## Discussion

The surveillance program implemented at Meyer Children's University Hospital during the first wave of the COVID-19 pandemic in Italy enabled the discovery of 13 positive cases in asymptomatic professionals (by 1,690 RT-PCR on NP swab), preventing possible outbreaks in the hospital setting. The occurrence of SARS-CoV-2 positivity on NP swabs performed in hospital workers (0.8%) was lower than Tuscan (4.4%) and Italian population means (6.8%) in the same period (April 2020). This result is in agreement with recent studies comparing HCWs and positivity incidences in patients proving the efficacy of prevention measures adopted by HCWs (18–20).

Although non-statistically significant, it was noticed that a trend of SARS-CoV-2 positivity was higher in non-COVID-19 areas. This paradoxical result could be explained by the fact that workers in COVID-19 wards follow stricter isolation and prevention protocols, both at work and home settings, and suggests that the consciousness of the risks associated with the infection is pivotal in COVID-19 prevention.

Healthcare workers have played a crucial role in fighting against COVID-19 during the pandemic, working hard and accepting the high risk of SARS-CoV-2 infection to which they were routinely exposed. To the present day, they still represent the primary resource each country has to face during the COVID-19 pandemic. Therefore, their surveillance needs to be guaranteed as a priority by every government. At the same time, workers should contribute to the process by strictly following safety indications and behaving to minimize transmission of SARS-CoV-2. A local policy regulating the latter factors appears to be of paramount importance to encourage an effective containment of SARS-CoV-2 spread. In addition, HCWs should be conscious of the importance of self-monitoring, and self-care is being concerned about clinical manifestations that may indicate SARS-CoV-2 infection even when they suffer from mild symptoms.

A notable outcome of the present study is that residents resulted in the less affected working role within the category of patient-facing health providers (which also includes doctors, nurses, intermediate care technicians, and allied health professionals). We may suppose there exist several reasons for this peculiar result: first, the organized shifts allowed to reduce the probabilities of contact for residents; besides, the most significant part of them, coming from other Italian cities, remained in Florence all along the lockdown period, isolated from their relatives and friends. Conversely, it seems reasonable that nurses accounted for the most of total positive cases. This may be due, despite appropriate safety measures, to their being in potential contact with patients for a longer period of time compared with all the other working roles.

We acknowledge some limitations in the study. Indeed, we did not ask about the type of personal protective equipment used by the workers; however, specific indications were in place in the hospital. Furthermore, we were not able to collect specific data about the frequency and duration of their exposure to hospitalized COVID-19 patients; however, among workers in COVID-19 areas, no RT-PCR positivity was registered with the lowest serological positivity rate.

On the contrary, we carried out an extensive screening program, which was possible thanks to considerable laboratory capacity together with the collaboration of the personnel, and it may not be available at every healthcare facility. Thus, our peculiar setting allowed us to screen and monitor all workers of the hospital regardless of the presence of clinical manifestations and the level of working exposure risk. In addition, the surveillance program succeeded to cover several aims, i.e., screening, monitoring of contacts, or before re-admissions at work after recovering from an acute influenza-like -illness.

The following suggestions result from the data studies: first, the risk for a worker who recovered from an influenza-like illness to be still positive and infectious for SARS-CoV-2 is significantly high and suggest that the decision of the hospital to test all workers before re-admission at work may be wise and relevant. Second, we observed that a screening based on the serological test alone, missing almost 50% of infectious workers, is not useful to reduce virus spread in an environment as the healthcare setting. Third, as already highlighted by other studies (5, 24–26), accurate surveillance is essential to reduce virus spread among HCWs, patients, and the community and to limit the shortage of skilled professionals. We may assume that screening programs can help HCWs to preserve their health and effectiveness at work and can sensitize them to be the protagonists in avoiding SARS-CoV-2 infection. Indeed, the first screening experience was followed by the “*Uffa project”* with self-collected nasal swabs, which showed to be a feasible and well-tolerated procedure to the SARS-CoV-2 screening program in healthcare system with a high adherence by workers from our hospital (27). The implementation of surveillance through an efficient screening program offered to all professionals may be wise and relevant.

## Data Availability Statement

The raw data supporting the conclusions of this article will be made available by the authors, without undue reservation.

## Ethics Statement

The studies involving human participants were reviewed and approved by Pediatric Ethic Committee Tuscany Region. The patients/participants provided their written informed consent to participate in this study.

## Author Contributions

CA conceptualized the work. VG, MM, and MG have drafted the manuscript. VG, MM, MG, LL, SR, LP, PB, CB, GI, AZ, and CA performed the investigations and critically revised the manuscript. All authors approved the final version of the manuscript as submitted and agreed to be accountable for all aspects of the work.

## Conflict of Interest

The authors declare that the research was conducted in the absence of any commercial or financial relationships that could be construed as a potential conflict of interest.

## Publisher's Note

All claims expressed in this article are solely those of the authors and do not necessarily represent those of their affiliated organizations, or those of the publisher, the editors and the reviewers. Any product that may be evaluated in this article, or claim that may be made by its manufacturer, is not guaranteed or endorsed by the publisher.

## Collaborators

Valeria Astorino, Silvia Boscia, Elisa Calistri, Francesco Citera, Martina Cortimiglia, Sara Guiducci, Giusi Mangone, Francesco Nieddu, Caterina Pelosi, Giancarlo Perferi and Claudia Valleriani.
